# A novel *BCL11A* polymorphism influences gene expression, therapeutic response and epilepsy risk: A multicenter study

**DOI:** 10.3389/fnmol.2022.1010101

**Published:** 2022-12-09

**Authors:** Shitao Wang, Xuemei Cai, Shiyong Liu, Qixin Zhou, Ting Wang, Sunbing Du, Dan Wang, Fei Yang, Qian Wu, Yanbing Han

**Affiliations:** ^1^Department of Neurology, First Affiliated Hospital of Kunming Medical University, Kunming, China; ^2^Department of Neurology, Affiliated Fuyang People's Hospital of Anhui Medical University, Fuyang, China; ^3^Department of Clinical Laboratory, First Affiliated Hospital of Kunming Medical University, Kunming, China; ^4^Department of Neurosurgery, Xinqiao Hospital, Army Medical University (Third Military Medical University), Chongqing, China; ^5^Laboratory of Learning and Memory, Kunming Institute of Zoology, Chinese Academy of Sciences, Kunming, China

**Keywords:** *BCL11A*, polymorphism, epilepsy, gene, drug resistance

## Abstract

**Background:**

Genetic factors have been found to be associated with the efficacy and adverse reactions of antiseizure medications. *BCL11A* is an important regulator of the development of neuronal networks. However, the role of *BCL11A* in epilepsy remains unclear. This study aimed to evaluate the genetic association of *BCL11A* with the susceptibility to develop epileptic seizures and therapeutic response of patients with epilepsy in Han Chinese.

**Methods:**

We matched 450 epilepsy cases with 550 healthy controls and 131 drug-resistant epilepsy patients with 319 drug-responsive epilepsy patients from two different centers. Genetic association analysis, genetic interaction analysis, expression quantitative trait loci analysis and protein–protein interaction analysis were conducted.

**Results:**

Our results showed that rs2556375 not only increases susceptibility to develop epileptic seizures (OR = 2.700, 95% = 1.366–5.338, *p* = 0.004 and OR = 2.984, 95% = 1.401–6.356, *p* = 0.005, respectively), but also increases the risk of drug resistance(OR = 21.336, 95%CI =2.489–183.402, *p* = 0.005). The interaction between rs2556375 and rs12477097 results in increased risk for pharma coresistant. In addition, rs2556375 regulated *BCL11A* expression in human brain tissues (*p* = 0.0096 and *p* = 0.033, respectively). Furthermore, the protein encoded by *BCL11A* interacted with targets of approved antiepileptic drugs.

**Conclusion:**

*BCL11A* may be a potential therapeutic target for epilepsy. Rs2556375 may increase the risks of epilepsy and drug resistance by regulating *BCL11A* expression in human brain tissues. Moreover, the interaction between rs2556375 and rs12477097 results in increased risk for drug resistance.

## Introduction

Epilepsy is a neurological disease with genetic factors in its pathogenesis (International League Against Epilepsy Consortium on Complex Epilepsies, 2014). The heritability of epilepsy is estimated to be more than 50% of all epilepsies (Pal et al., 2010). So far, a large number of genes associated with epilepsy has been identified with the help of advances in genetic technologies (Wang et al., 2017). However, evidence for the associations of some of these genes with epilepsy is controversial partly due to study power and scope (Cavalleri et al., 2007; Kasperaviciūte et al., 2010; Guo et al., 2012; Leu et al., 2012; Steffens et al., 2012; Franco and Perucca, 2015). Genetic factors have been found to be associated with the efficacy and adverse reactions of antiepileptic drugs (Franco and Perucca, 2015; Balestrini and Sisodiya, 2018). Currently, approximately one-third of epileptic seizures cannot be effectively controlled with available antiepileptic drugs, so the discovery of new drug targets has become increasingly important.

The BCL11A gene is expressed at high levels in hematopoietic tissues and the brain (Nakamura et al., 2000; Funnell et al., 2015). Functional deficits in *BCL11A* can result in persistence of HbF (Satterwhite et al., 2001; Sankaran et al., 2008). BCL11A-L is an important protein product of BCL11A gene. In hippocampal neurons, downregulation of BCL11A-L expression upregulates axon branching and dendrite outgrowth and increases the arbor complexity of axons and dendrites, while overexpression of BCL11A-L reduces neurite arborization (Kuo et al., 2009). BCL11A-L may regulate deleted in colorectal cancer and microtubule-associated protein 1b expression through an interaction with calmodulin-dependent serine protein kinase, thereby limiting neuritogenesis (Kuo et al., 2010). Changes in the expression of *BCL11A* will lead to abnormal development of neurons, which can cause abnormal discharge of neurons, resulting in seizures. To date, *BCL11A* mutations have been shown to be associated with intellectual disability syndrome (Dias et al., 2016; Cai et al., 2017; Soblet et al., 2018). Two *de novo* BCL11A heterozygous variants were reported to be associated with patients with epileptic encephalopathy (Yoshida et al., 2018), and it also indicated that a *de novo* mutation in the *BCL11A* gene result in epilepsy (Korenke et al., 2020). However, the role of common *BCL11A* single nucleotide polymorphisms(SNPs) in therapeutic response of patients with epilepsy has not been evaluated, and the role of common *BCL11A* SNPs in epilepsy in Han Chinese remains unknown.

In this study, we analyzed *BCL11A* SNPs to explore their potential associations with epilepsy risk and therapeutic response of patients with epilepsy, and then performed expression quantitative trait loci (eQTL) analysis to investigate effects of identified SNPs on *BCL11A* expression. Furthermore, protein–protein interaction(PPI) analysis was performed to further evaluate the role of *BCL11A* in epilepsy treatment.

## Materials and methods

### Study population

We recruited 450 epilepsy cases diagnosed at the First Affiliated Hospital of Kunming Medical University and Xinqiao Hospital. In parallel, we recruited 550 healthy controls without a personal history of epilepsy or a family history of epilepsy for routine health checkups from the same hospitals. All samples were stored in the biobanks of the First Affiliated Hospital of Kunming Medical University and Kunming Medical University. The diagnoses of epilepsy and drug-resistant epilepsy are based on 2017 and 2010 International League Against Epilepsy criteria, respectively. Carbamazepine, valproic acid, levetiracetam and Lamotrigine were prescribed to the epilepsy patients. Symptomatic epilepsy was ruled out through blood tests, disease history review, imaging examination, etc. All participants were required to sign the informed consent form.

### Selection and genotyping of genetic polymorphisms

*BCL11A* Tagging SNPs with a minor allele frequency greater than 0.05 in Han Chinese were selected from the 1,000 Genomes database^1^ using Haploview software. The Tissue DNA Kit (OMEGA, United States) and the Blood DNA Mini Kit (OMEGA, United States) were used to extract genomic DNA from brain tissues and peripheral blood, respectively. Brain tissue samples was derived from abnormal discharge lesions in the temporal lobe of patients with drug-resistant epilepsy patients undergoing epilepsy surgery. Genotyping was conducted using the Bio-Rad CFX96 (BioRad, United States) platform, and the primers were designed with Primer Premier V6.0 (Premier Biosoft Inc., United States). Details on PCR primers are provided in Supplementary Table 1.

### Functional analysis

We performed eQTL analysis using 16 brain tissues from patients with drug-resistant epilepsy to investigate whether identified SNPs regulate *BCL11A* expression in human brain tissues. Tissue RNA Kit (Omega, United States) and the FastQuant cDNA kit (Tiangen, China) are used for genomic RNA extraction and cDNA reverse transcription, respectively, and the SYBR^®^Green I (Vazyme, China) and the ABI QuantStudio 6 Flex^™^ (ABI, United States) analyzer are used for Quantitative PCR. We designed the primers with Primer Premier V6.0 (Premier Biosoft Inc., United States). Details on Quantitative PCR premiers are provided in Supplementary Table 2. Functional effect of identified SNPs on *BCL11A* expression was further verified by data from the Genotype-Tissue Expression project (GTEx).^2^

### Further evaluation of *BCL11A* in epilepsy treatment

To further evaluate the role of *BCL11A* in epilepsy treatment, we obtained approved antiepileptic drug targets from DrugBank5.0 (Wishart et al., 2018) and the Therapeutic Target Database 2020 (Wang et al., 2020). The interactions between the protein encoded by *BCL11A* and approved antiepileptic drug targets were evaluated with Cytoscape V.3.7.2 (Su et al., 2014).

### Statistical methods

Associations of *BCL11A* SNPs with epilepsy and therapeutic response were analyzed with binary logistic regression. Hardy–Weinberg equilibrium and difference in gender were assessed using the Chi-square (χ^2^) test and Fisher’s exact test. The eQTL analysis and difference in mean age were analyzed by independent samples t-test. A *p* value <0.05 (two-tailed) was considered statistically significant. For Bonferroni correction, A p value <0.0056 (0.05/9) was considered statistically significant. All analyses were conducted with SPSS23.0(IBM Corp, Armonk, NY) and Graphpad Prism9.0.^3^

## Results

### Characteristics of populations

The baseline characteristics are summarized in Table 1.

**Table 1:** Characteristics of the patients and controls.

Characteristics	Epilepsy	Control	*P* ^a^	Nonresponders	Responders	*P* ^b^
Males/Females	214/236	244/306	0.314	57/74	157/162	0.271
Age(Mean ± SD)	24.63 ± 16.221	25.77 ± 15.664	0.260	28.74 ± 17.162	22.95 ± 15.535	0.001

SD: standard deviation.

Difference in gender between cases and controls analyzed using the χ^2^ test.

Difference in mean age analyzed using the independent sample *t*-test.

a *P-*values were calculated between epilepsy and control.

b *P-*values were calculated between nonresponders and responders.

### Association of *BCL11A* SNPs with epilepsy and therapeutic response

We selected nine tagging SNPs with a minor allele frequency greater than 0.05 from the 1,000 Genomes database (see Footnote 1) using Haploview software, which complied with the Hardy–Weinberg equilibrium in the control group(*p* > 0.05 for all; Tables 2, 3).

**Table 2:** Associations between *BCL11A* SNP genotypes and epilepsy risk.

SNP ID	Genotype	Epilepsy N(%)	Control N(%)	OR (95% CI)	*P* ^a^	*P* ^b^
rs356977	GG	259(57.6)	314 (57.1)	1.000	-	0.774
	GA	166 (36.9)	209 (38.0)	1.131 (0.711–1.798)	0.603	-
	AA	25 (5.6)	27 (4.9)	1.430 (0.739–2.770)	0.289	-
	GA + AA	191 (42.4)	236 (42.9)	1.184 (0.756–1.854)	0.460	-
rs2556375	TT	227 (50.4)	287 (52.2)	1.000	-	0.957
	TG	182 (40.4)	218 (39.6)	2.700 (1.366–5.338)	0.004	-
	GG	41 (9.1)	45 (8.2)	2.984 (1.401–6.356)	0.005	-
	TG + GG	223 (49.6)	263 (47.8)	2.774 (1.420–5.417)	0.003	-
rs6747099	GG	338 (75.1)	415 (75.5)	1.000	-	0.387
	GC	109 (24.2)	119 (21.6)	1.266 (0.894–1.793)	0.184	-
	CC	3 (0.7)	16 (2.9)	0.275 (0.077–0.980)	0.046	-
	GC + CC	112 (24.9)	135 (24.5)	1.158 (0.823–1.631)	0.399	
rs7577014	AA	267 (59.3)	323 (58.7)	1.000	-	0.720
	AG	165 (36.7)	190 (34.5)	1.265 (0.819–1.955)	0.289	-
	GG	18 (4.0)	37 (6.7)	0.728 (0.375–1.413)	0.348	-
	AG + GG	183 (40.7)	227 (41.3)	1.137 (0.749–1.727)	0.547	
rs10184550	GG	327 (72.7)	381 (69.3)	1.000	-	0.999
	GA	109 (24.2)	153 (27.8)	0.848 (0.596–1.204)	0.356	-
	AA	14 (3.1)	16 (2.9)	1.140 (0.529–2.458)	0.739	-
	GA + AA	123 (27.3)	169 (30.7)	0.875 (0.622–1.231)	0.442	-
rs10189857	GG	261 (58.0)	314 (57.1)	1.000	-	0.891
	GA	162 (36.0)	199 (36.2)	1.125 (0.708–1.789)	0.618	-
	AA	27 (6.0)	37 (6.7)	1.083 (0.583–2.011)	0.801	-
	GA + AA	189 (42.0)	236 (42.9)	1.115 (0.715–1.737)	0.631	-
rs12477097	CC	253 (56.2)	298 (54.2)	1.000	-	0.127
	CA	166 (36.9)	230 (41.8)	0.853 (0.519–1.403)	0.532	-
	AA	31 (6.9)	22 (4.0)	1.874 (0.943–3.725)	0.073	-
	CA + AA	197 (43.8)	252 (45.8)	0.990 (0.611–1.604)	0.967	-
rs12623979	CC	286 (63.6)	348 (63.3)	1.000	-	0.857
	CA	150 (33.3)	183 (33.3)	1.134 (0.774–1.661)	0.519	-
	AA	14 (3.1)	19 (3.5)	1.102 (0.517–2.349)	0.802	-
	CA + AA	164 (36.4)	202 (36.7)	1.130 (0.779–1.640)	0.520	-
rs13018474	GG	331 (73.6)	394 (71.6)	1.000	-	0.669
	GA	109 (24.2)	138 (25.1)	1.003 (0.708–1.423)	0.985	-
	AA	10 (2.2)	18 (3.3)	0.764 (0.336–1.742)	0.523	-
	GA + AA	119 (26.4)	156 (28.4)	0.977 (0.695–1.372)	0.892	-

SNP: Single nucleotide polymorphism, OR: Odds ratio, 95% CI: 95% confidence interval.

a *p-*values were calculated by logistic regression analysis with adjustment for gender and age.

b *P-*values were calculated using χ2 test for Hardy–Weinberg equilibrium in healthy control.

**Table 3:** Associations between *BCL11A* SNP genotypes and therapeutic response of patients with epilepsy.

SNP ID	Genotype	Nonresponders N(%)	Responders N(%)	OR (95% CI)	*P* ^a^	*P* ^b^
rs356977	GG	78 (59.5)	181 (56.7)	1.000	–	0.997
	GA	48 (36.6)	118 (37.0)	0.569 (0.284–1.136)	0.110	–
	AA	5 (3.8)	20 (6.3)	0.306 (0.097–0.968)	0.044	–
	GA + AA	53 (40.5)	138 (43.3)	0.525 (0.265–1.036)	0.063	–
rs2556375	TT	58 (44.3)	169 (53.0)	1.000	–	0.316
	TG	46 (35.1)	136 (42.6)	4.626 (0.579–36.995)	0.149	–
	GG	27 (20.6)	14 (4.4)	21.336 (2.489–183.402)	0.005	–
	TG + GG	73 (55.7)	150 (47.0)	6.633 (0.831–52.949)	0.074	–
rs6747099	GG	95 (72.5)	243 (76.2)	1.000	–	0.230
	GC	34 (26.0)	75 (23.5)	1.060 (0.601–1.870)	0.840	–
	CC	2 (1.5)	1 (0.3)	3.871 (0.335–44.759)	0.278	–
	GC + CC	36 (27.5)	76 (23.8)	1.104 (0.630–1.934)	0.730	–
rs7577014	AA	75 (57.3)	192 (60.2)	1.000	–	0.876
	AG	51 (38.9)	114 (35.7)	1.083 (0.550–2.135)	0.817	–
	GG	5 (3.8)	13 (4.1)	0.750 (0.228–2.464)	0.635	–
	AG + GG	56 (42.7)	127 (39.8)	1.040 (0.533–2.028)	0.908	–
rs10184550	GG	98 (74.8)	229 (71.8)	1.000	–	0.849
	GA	29 (22.1)	80 (25.1)	0.707 (0.394–1.270)	0.246	–
	AA	4 (3.1)	10 (3.1)	0.642 (0.187–2.208)	0.482	–
	GA + AA	33 (25.2)	90 (28.2)	0.699 (0.398–1.229)	0.213	–
rs10189857	GG	74 (56.5)	187 (58.6)	1.000	–	0.245
	GA	39 (29.8)	123 (38.6)	0.672 (0.329–1.371)	0.275	–
	AA	18 (13.7)	9 (2.8)	3.285 (1.200–8.989)	0.021	–
	GA + AA	57 (43.5)	132 (41.4)	0.914 (0.459–1.820)	0.798	–
rs12477097	CC	69 (52.7)	184 (57.7)	1.000	–	0.997
	CA	49 (37.4)	117 (36.7)	0.957 (0.440–2.083)	0.911	–
	AA	13 (9.9)	18 (5.6)	2.849 (1.051–7.727)	0.040	–
	CA + AA	62 (47.3)	135 (42.3)	1.193 (0.558–2.550)	0.648	–
rs12623979	CC	88 (67.2)	198 (62.1)	1.000	–	0.780
	CA	40 (30.5)	110 (34.5)	0.550 (0.301–1.005)	0.052	–
	AA	3 (2.3)	11 (3.4)	0.345 (0.088–1.359)	0.128	–
	CA + AA	43 (32.8)	121 (37.9)	0.528 (0.292–0.956)	0.035	–
rs13018474	GG	104 (79.4)	227 (71.2)	1.000	–	0.964
	GA	24 (18.3)	85 (26.6)	0.464 (0.256–0.842)	0.012	–
	AA	3 (2.3)	7 (2.2)	0.560 (0.136–2.312)	0.423	–
	GA + AA	27 (20.6)	92 (28.8)	0.473 (0.266–0.842)	0.011	–

SNP: Single nucleotide polymorphism, OR: Odds ratio, 95% CI: 95% confidence interval.

a *p*-values were calculated by logistic regression analysis with adjustment for gender and age.

b *P-*values were calculated using χ2 test or Fisher’s exact test for Hardy–Weinberg equilibrium in drug-responsive epilepsy patients.

Of the nine tagging SNPs, rs2556375 is associated with increased risk of epilepsy (OR = 2.700, 95% = 1.366–5.338, *p* = 0.004 and OR = 2.984, 95% = 1.401–6.356, *p* = 0.005, respectively) after adjusting for gender and age and Bonferroni correction at genotypic level (Table 2). In addition, the rs6747099 genotype distribution was also different between the epilepsy and control groups after adjusting for gender and age(OR = 0.275, 95%CI = 0.077–0.980, *p* = 0.046; Table 2), but did not reach significance after Bonferroni correction(*p* > 0.0056).

Similarly, rs2556375 is associated with increased risk of drug resistance (OR = 21.336, 95%CI = 2.489–183.402, *p* = 0.005) after adjusting for gender and age at genotypic level (Table 3). At the same time, we also found that rs356977, rs10189857, rs12477097 and rs13018474 are associated with drug resistance after adjusting for gender and age(OR = 0.306, 95%CI = 0.097–0.968, *p* = 0.044; OR = 3.285, 95%CI = 1.200–8.989, *p* = 0.021; OR = 2.849, 95%CI = 1.051–7.727, *p* = 0.040 and OR = 0.464, 95%CI = 0.256–0.842, *p* = 0.012, respectively) at genotypic level (Table 3). However, only rs2556375 reached significance after Bonferroni correction (*p* < 0.0056).

### Genetic interaction analyses on epilepsy risk and therapeutic response

**Figure 1:**
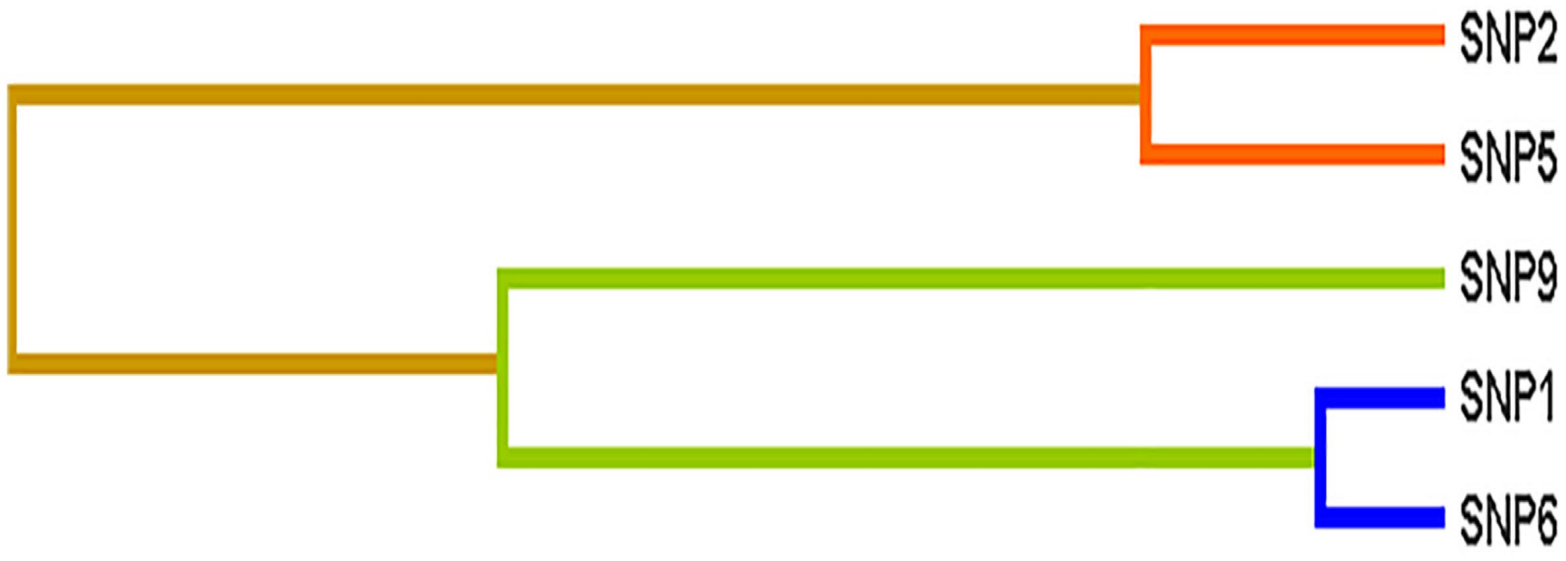
Different types of interaction dendrogram among nine SNPs. SNP1: rs12477097, SNP2: rs12623979, SNP5: rs356977, SNP6: rs2556375, SNP9: rs7577014.

The interactions among nine SNPs were detected by multifactor dimensionality reduction(MDR). For the association with epilepsy risk, no significant model was detected by MDR. For the association with therapeutic response, Table 4 provided the results testing by MDR. Two-to three-locus models were significant (*p* = 0.0107). An interaction dendrogram showed the strongest interaction between rs2556375 and rs12477097 (Figure 1).

**Table 4:** MDR analysis of *BCL11A* SNPs showed different interactions for therapeutic response of patients with epilepsy.

Model	Training Bal.Acc.	Testing Bal.Acc.	Sign Test(*p*)	CV Consistence
SNP6	0.5811	0.5813	10 (0.0010)	10/10
SNP1SNP6	0.6133	0.5445	9 (0.0107)	6/10
SNP1SNP2SNP6	0.6424	0.5344	9 (0.0107)	4/10
SNP1SNP5SNP6SNP9	0.6756	0.5548	9 (0.0107)	7/10

SNP1: rs12477097, SNP2: rs12623979, SNP5: rs356977, SNP6: rs2556375, SNP9: rs7577014.

To obtain the OR and 95%CI for a joint effect of rs2556375 and rs12477097, we conducted genetic interaction analysis by using logistic regression analyses. As shown in Table 5, the participants with rs12477097 AC genotype and rs2556375 GG genotype had higher risk of drug resistance than the participants with rs12477097 CC genotype and rs2556375TT genotype.

**Table 5:** Joint effects analysis of rs2556375 and rs12477097 for therapeutic response of patients with epilepsy.

Types	Nonresponders	Responders	P	OR (95% CI)
CC/TT	32	115	–	1
CC/GT	28	60	0.076	1.733 (0.945–3.179)
CC/GG	9	9	0.010	3.868 (1.387–10.784)
AC/TT	20	49	0.315	1.402 (0.725–2.710)
AC/GT	17	64	0.992	1.004 (0.510–1.976)
AC/GG	12	4	0.000	11.841 (3.447–40.680)
AA/TT	6	5	0.062	3.398 (0.942–12.259)
AA/GT	1	12	0.282	0.318 (0.039–2.570)
AA/GG	6	1	0.006	20.583 (2.342–180.871)

*p*-values were calculated by logistic regression analysis with adjustment for gender and age.

### Functional analysis

**Figure 2:**
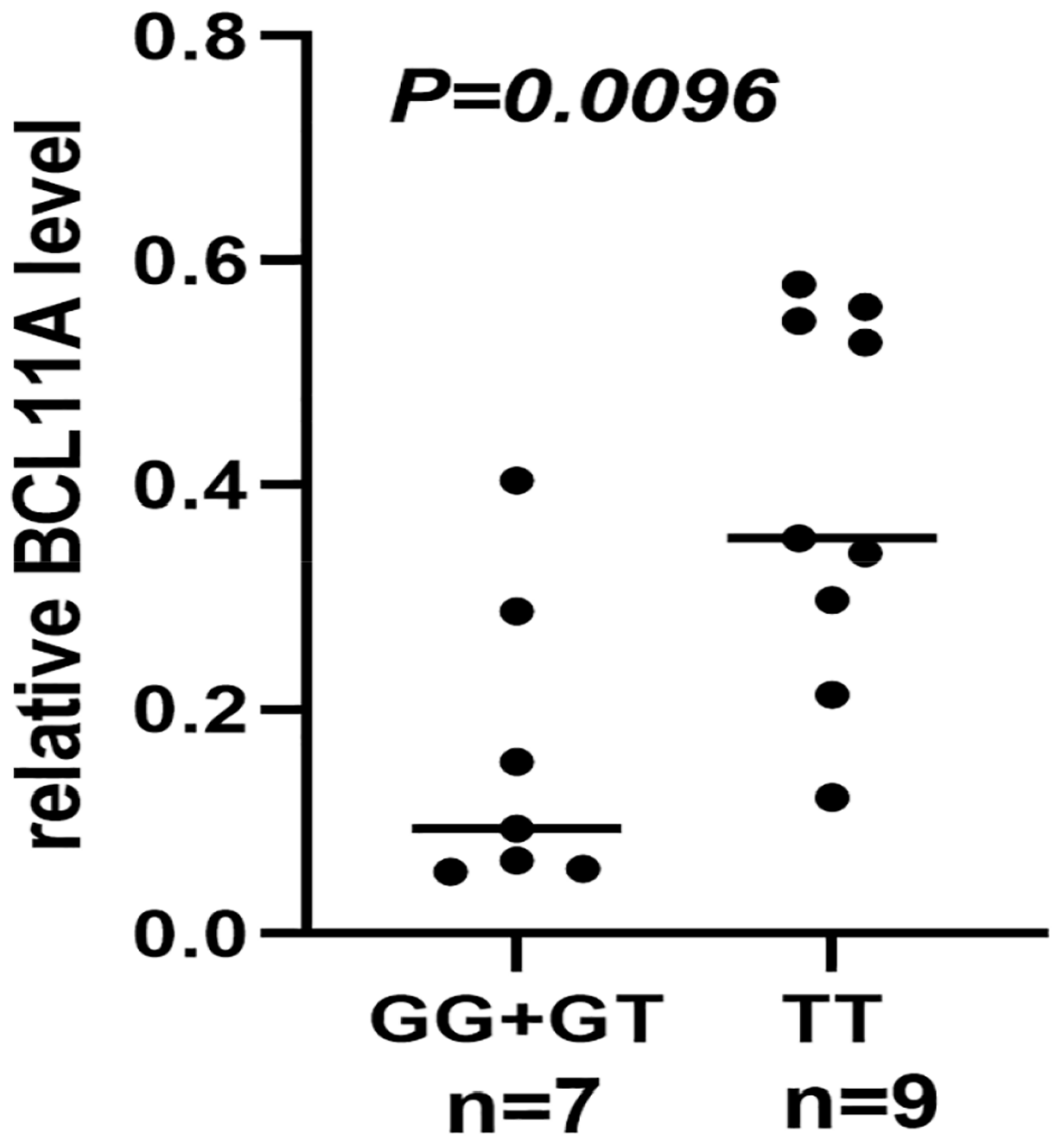
The rs2556375 is an eQTL in brain tissue of drug-resistant epilepsy patients. The carriers of the G allele exhibited downregulated BCL11A gene expression.

**Figure 3:**
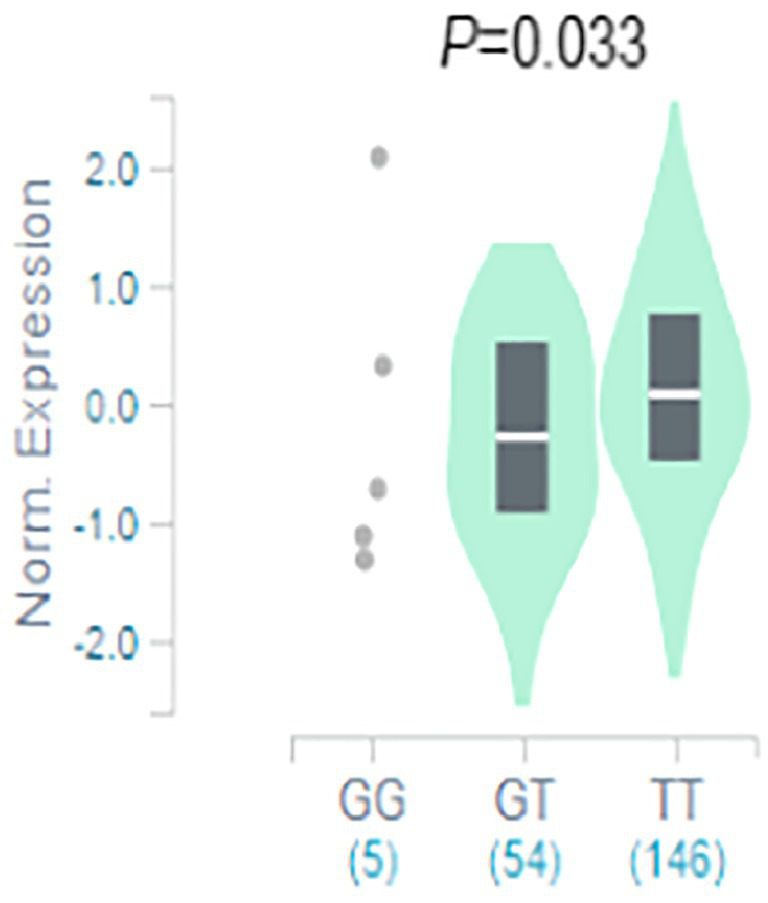
The rs2556375 is an eQTL in cortex. Data were retrieved from the brain tissue database GTEx (https://gtexportal.org/).

The result of functional analysis showed that rs2556375 is an eQTL in drug-resistant epileptic brain tissue, and the carriers of the G allele exhibited downregulated *STX1B* gene expression (Figure 2). Furthermore, this finding was confirmed by data from the brain tissue database GTEx (Figure 3).^5^

### Further evaluation of *BCL11A* in the treatment of epilepsy

**Figure 4:**
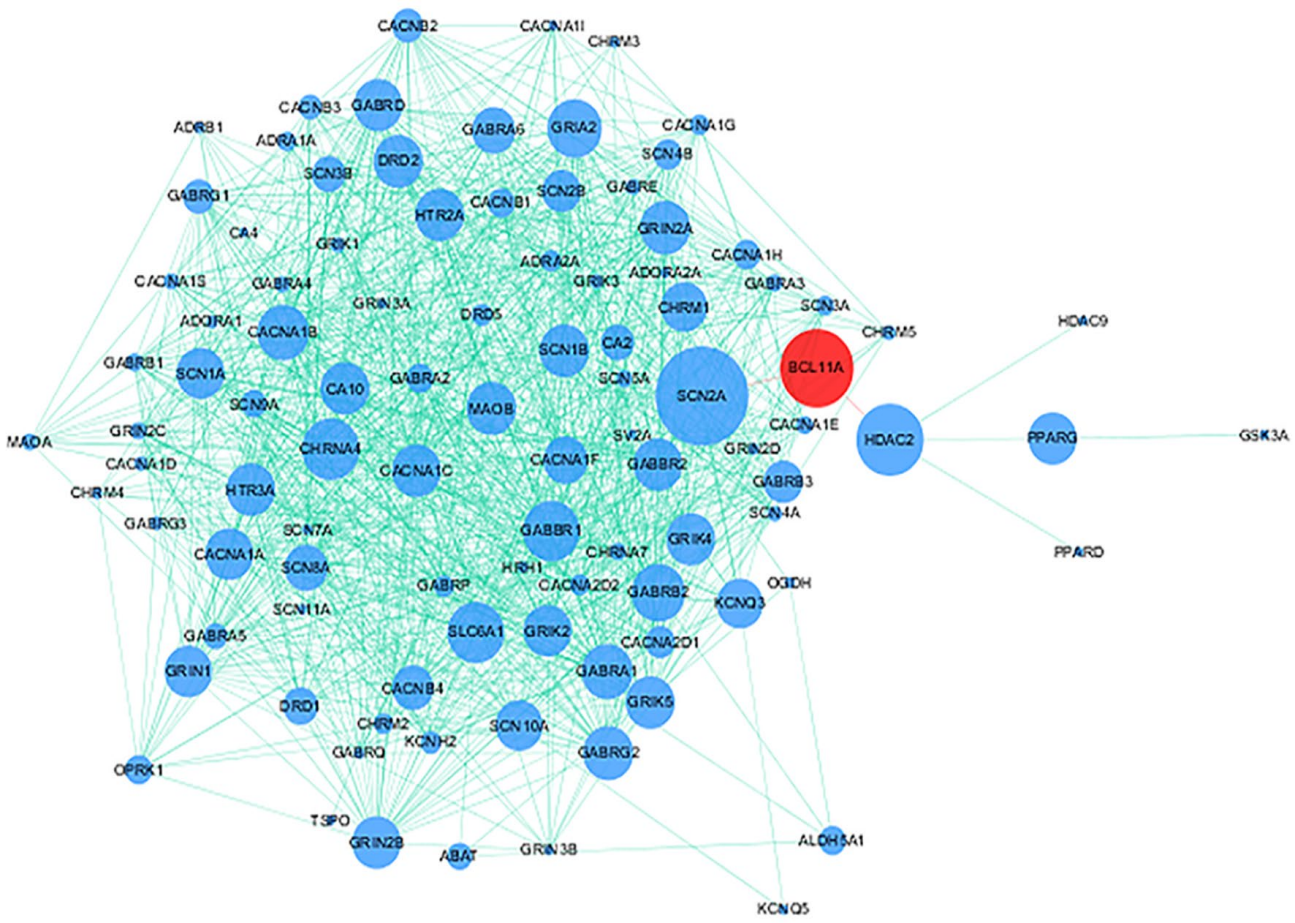
Protein–protein interaction (PPI) network of BCL11A gene and genes targeted by approved antiepileptic drugs. Red node and blue nodes represent BCL11A gene and genes targeted by approved antiepileptic drugs, respectively. The proteins connected by the red line mean that these proteins have direct interaction.

115 genes targeted by approved antiepileptic drugs were obtained from DrugBank5.0 (Wishart et al., 2018) and the Therapeutic Target Database 2020 (Wang et al., 2020). Details on 115 genes are provided in Supplementary Table 3. PPI analysis showed that the protein encoded by *BCL11A* interacts with approved antiepileptic drug targets (Figure 4).

## Discussion

*BCL11A* is an important regulator of the development of neuronal networks, and its proper expression is very important for axon branching and dendrite growth (Kuo et al., 2009, 2010). Based on the above results, we speculate that *BCL11A* may affect the risks of epilepsy and drug resistance by regulating the development of neuronal networks.

Gene SNPs have been found to be associated with the risk of diseases and the efficacy of drug treatment (Tawfeek and Alhassanin, 2018; Ebid et al., 2020; Pozhidaev et al., 2020; de Assis et al., 2021). However, the association between the common *BCL11A* SNPs and epilepsy and the association between the common *BCL11A* SNPs and therapeutic response of patients with epilepsy in Han Chinese remain unclear. In this study, by conducting genetic association analysis, genetic interaction analysis, eQTL analysis and PPI analysis, we have identified a novel SNP (rs2556375) associated with epilepsy and therapeutic response of patients with epilepsy. To our knowledge, this is the first study that evaluate the associations of common *BCL11A* SNPs with epilepsy risk and therapeutic response of patients with epilepsy in Han Chinese.

We performed a two-stage case–control study to evaluate the associations between *BCL11A* SNPs and epilepsy. In the Stage I study, we found that rs2556375 increases epilepsy risk (Table 2). In the Stage II study, further analyses suggested that rs2556375 increases the risk of drug resistance (Table 3). Furthermore, we also found that the participants with rs12477097 AC genotype and rs2556375GG genotype had higher risk of drug resistance than the participants with rs12477097 CC genotype and rs2556375TT genotype (Table 5), suggesting an important pathogenic mechanism that rs2556375 affects the risk of drug resistance by interacting with rs12477097. Combined with the above findings, we can reasonably speculate that rs2556375 may play an important role in the risk and therapeutic response of patients with epilepsy.

Among all the tagging SNPs, rs2556375, rs6747099, rs356977, rs10189857, rs12477097and rs13018474 in *BCL11A* also showed associations with epilepsy or therapeutic response of patients with epilepsy, but the association did not exist after Bonferroni correction. However, the role of these SNPs in the risk and therapeutic response of patients with epilepsy could not be simply ignored, as further verification of our findings is necessary. Although we did not find that rs12477097 alone affected therapeutic response of patients with epilepsy, we found that the interaction between rs2556375 and rs12477097 could affect therapeutic response of patients with epilepsy.

Previous studies have found that gene SNPs often affect the risk of diseases by regulating gene expression (Maurano et al., 2012; Wang et al., 2021a,b). To clarify how rs2556375 affects the risks of epilepsy and drug resistance, we included 16 brain tissues from patients with drug-resistant epilepsy, and further used data from GTEx database for eQTL analysis to evaluate whether rs2556375 regulates the expression level of *BCL11A* in human brain tissue. Our results showed that rs2556375 regulates the expression of *BCL11A* in human brain tissue, especially in brain tissue of patients with drug-resistant epilepsy (Figure 2), which further demonstrates what we found in genetic association studies. Furthermore, the results of PPI analysis verify the role of BCL11A in epilepsy treatment. These above results will contribute to the discovery of new therapeutic targets for epilepsy.

Although we conducted genetic association analysis, genetic interaction analysis, eQTL analysis and PPI analysis and found that the *BCL11A* may be associated with the risk of epilepsy and therapeutic response of patients with epilepsy in Han Chinese. However, the sample size included in this study is relatively small, and we only performed functional analysis in human brain tissue. Therefore, it is necessary to performed further research with large samples and functional analysis to verify our conclusions.

## Conclusion

*BCL11A* may be a potential therapeutic target for epilepsy. Rs2556375 may increase the risks of epilepsy and drug resistance by regulating *BCL11A* expression in human brain tissues. Furthermore, the interaction between rs2556375 and rs12477097 results in increased risk for drug resistance. These findings will facilitate translation of genetic findings to clinical treatment in the future.

## Data availability statement

The original contributions presented in this study are included in the article/Supplementary material, further inquiries can be directed to the corresponding author.

## Ethics statement

The studies involving human participants were reviewed and approved by Ethics Committee of the First Affiliated Hospital of Kunming Medical University. Written informed consent to participate in this study was provided by the participants’ legal guardian/next of kin.

## Author contributions

YH and SW contributed to study design. SD, TW, and DW performed the experiments and contributed to the data collection. QW, XC, and FY performed the statistical analysis. YH, SW, QZ, and SL were involved in the writing of the manuscript. All authors contributed to the article and approved the submitted version.

## Funding

This work was supported by the National Natural Science Foundation of China (81660228 and 81601134), Yunnan Province Talent Training Program (2017HB048), Yunnan Health Training Project of High Level Talents (L-2019019 and H-2018056), and Yunnan Science and Research Funding Program (2016NS029).

## Conflict of interest

The authors declare that the research was conducted in the absence of any commercial or financial relationships that could be construed as a potential conflict of interest.

## Publisher’s note

All claims expressed in this article are solely those of the authors and do not necessarily represent those of their affiliated organizations, or those of the publisher, the editors and the reviewers. Any product that may be evaluated in this article, or claim that may be made by its manufacturer, is not guaranteed or endorsed by the publisher.

## Footnotes

^1^ http://www.internationalgenome.org/

^2^ http://www.gtexportal.org/home/

^3^ www.graphpad-prism.cn

^5^ https://gtexportal.org/
