# Supplementary material for: A novel BCL11A polymorphism influences gene expression, therapeutic response and epilepsy risk: A multicenter study
Source: Front Mol Neurosci. 2022 Dec 9;15:1010101. doi: 10.3389/fnmol.2022.1010101 (PMC9780294; doi:10.3389/fnmol.2022.1010101)
Supplement: Supplementary file 1 [file Data_Sheet_1.doc]

**Table S1** PCR primers for the nine SNPs

| SNP ID | Allele |  | Primers |
| --- | --- | --- | --- |
| rs356977 | G>A | AF | TCTTGTGCCCCAGTCCTCCCTTAAA |
|  |  | GF | TCTTGTGCCCCAGTCCTCCCTTAAG |
|  |  | R | ACGTCAAGGGTTAAGCAGCCTTCCC |
| rs2556375 | T>G | GF | AAAAAGAAAATTCCTTGGCATAAGG |
|  |  | TF | AAAAAGAAAATTCCTTGGCATAAGT |
|  |  | R | TCTGAAATTTGATCAGCAAACATAT |
| rs6747099 | G>C | CF | AAGAACAAAGACATACGGGGTGATC |
|  |  | GF | AAGAACAAAGACATACGGGGTGATG |
|  |  | R | TAGGCAGAATTCCCTTTCTCTCCCC |
| rs7577014 | A>G | AF | ACCGTGAGCGCGCTGGTGTCCAAAA |
|  |  | GF | ACCGTGAGCGCGCTGGTGTCCAAAG |
|  |  | R | CGGGGAGAAAAGAGGTGAGACTGGC |
| rs10184550 | G>A | AF | AACTCAAGACTATCAGAATGATATA |
|  |  | GF | AACTCAAGACTATCAGAATGATATG |
|  |  | R | TTTGATAAGTATCTATACAAATATT |
| rs10189857 | G>A | AF | CCCTTGTTCTATCAGCAGGTCAAGA |
|  |  | GF | CCCTTGTTCTATCAGCAGGTCAAGG |
|  |  | R | AGAACAGCTTGTCACAGTTCTCTAC |
| rs12477097 | C>A | AF | TTCCCCTGCAGAATTAGACAGCTAA |
|  |  | CF | TTCCCCTGCAGAATTAGACAGCTAC |
|  |  | R | GCTTCTTAGGCTGATAGAAATGTTT |
| rs12623979 | C>A | AF | CTAGTCCCAGGAATGTCCTCCATGA |
|  |  | CF | CTAGTCCCAGGAATGTCCTCCATGC |
|  |  | R | TTCTGTTTTTGCAGTTAGCTTAAAG |
| rs13018474 | G>A | AF | TTGGGAGGCTGAGGCAGGAGAATCA |
|  |  | GF | TTGGGAGGCTGAGGCAGGAGAATCG |
|  |  | R | GCAACCTCTGCCTCCTAGGTTCAAG |

F: forward, R: reverse, SNP: Single nucleotide polymorphism

**Table S2**  QPCR primers for BCL11A gene

| Gene |  | Primers |
| --- | --- | --- |
| *BCL11A* | F: | GCCAGAGGATGACGATTGTT |
|  | R: | GCTCATCTTTACCTGCTATGTGTT |

F: forward, R: reverse

**Table S3** 115 genes targeted by approved antiepileptic drugs

| **Epilepsy drugs** | **Genes** |
| --- | --- |
| Phenobarbital, Primidone, phenytoin, Carbamazepin, Valproate, Clonazepam, Clobazam, Gabapentin, lamotrigine, Topiramate, Oxcarbazepine, Tiagabine, Levetiracetam, Zonisamide, Felbamate, Pregabalin, Vigabatrin. | NR1I2, GRIA2, GABRA1, CHRNA4, CHRNA7, GRIK2, GRIN1, GRIN2A, GRIN2B, GRIN2C, GRIN2D, GRIN3A, GRIN3B, GABRA3, GABRA6, GABRA5, GABRB2, GABRB3, GABRD, GABRE, GABRG1, GABRG2, GABRG3, GABRP, GABRQ, SCN5A, SCN3A, CACNA1C, CACNA1D, CACNA1F, CACNA1S, CACNB1, CACNB2, CACNB3, CACNB4, CACNA1A, SCN8A, KCNH2, SCN1A, SCN1B, SCN2A, SCN4A, SCN7A, SCN9A, SCN10A, SCN11A, ALDH5A1, HDAC2, PPARA, PPARD, OGDH, SCN2B, SCN3B, SCN4B, PPARG, HDAC9, ACADSB, TSPO, CACNA2D1, KCNQ5, ADORA1, CACNA1B, CACNA2D2, KCNQ3, HTR3A, CACNA1E, ADRA2A, HRH1, OPRK1, ADORA2A, ADRA1A, DRD2, GABRA4, ADRB1, DRD1, DRD5, GABRA2, GABRB1, CHRM1, CHRM2, CHRM3,CHRM4, CHRM5, HTR2A, CA2, GRIK1, GRIK3, GRIK4, GRIK5, CA3, CA4, SLC6A1, SV2A, CA5B,  CA10, CA11, CA12, CA13, MAOB, CA7, CA9, CACNA1H, CA1, CACNA1G, CA5A, CA6, CA8, CA14, CACNA1I, MAOA, ABAT, GABBR1, GSK3A, GABBR2, SLC5A6. |
